# Endometrial sampling devices for early diagnosis of endometrial lesions

**DOI:** 10.1007/s00432-016-2215-3

**Published:** 2016-08-11

**Authors:** Jiang Du, Yaling Li, Shulan Lv, Qing Wang, Chao Sun, Xin Dong, Ming He, Qurat Ulain, Yongxing Yuan, Xiaoqian Tuo, Nasra Batchu, Qing Song, Qiling Li

**Affiliations:** 1 Department of Obstetrics and Gynecology, First Affiliated Hospital, Xi’an Jiaotong University, Xi’an, 710061 Shaanxi China; 2 Gongzhuling Health Workers High School, Gongzhuling, 136100 Jilin China; 3 Cardiovascular Research Institute, Morehouse School of Medicine, Atlanta, GA 30310 USA; 4 Center of Big Data and Bioinformatics, First Affiliated Hospital, Xi’an Jiaotong University, Xi’an, 710061 Shaanxi China

**Keywords:** Endometrial lesions, Aspiration, Biopsy, Brush, Screening

## Abstract

**Purpose:**

Endometrial carcinoma is the most common gynecologic malignancy in both developed and some developing countries. Unlike cervical cancer, for which there is routine screening, only patients symptomatic for endometrial carcinoma typically seek medical help for its diagnosis and treatment. Dilatation and curettage (D&C) has been the standard procedure for evaluating suspicious endometrial lesions. The discomfort and injury caused by the D&C procedure, however, restrict its use as a screening method for early diagnosis of endometrial lesions. High-risk endometrial cancer patients would benefit from an effective and low-cost screening test. In recent years, several endometrial devices have been developed and proposed as screening tools.

**Methods:**

We have reviewed and evaluated the literature relating to the endometrial sampling devices in clinical use or clinical trials, with the goal of comparing devices and identifying the most appropriate ones for screening for endometrial lesions. Eligible literature was identified from systematic PubMed searches, and the relevant data were extracted. Comments, letters, unpublished data, conference proceedings, and case reports were excluded from our search. Seventy-four articles on endometrial sampling devices were obtained for this review.

**Results:**

The main screening devices for endometrial carcinoma are aspiration devices (such as the Vabra aspirator), Pipelle, Tao Brush, and SAP-1 device. Among these devices, the Tao Brush is the most promising endometrial sampler for screening for endometrial lesions. However, its sampling insufficiency, cost, and unsuccessful insertion rate (20 % in nulliparous and 8 % in parous women) are problematic.

**Conclusions:**

A more accurate and low-cost endometrial sampler, with improved specimen sufficiency and higher sensitivity for endometrial lesions, needs tobe developed and clinically verified.

## Introduction

Endometrial carcinoma is the most common gynecologic malignancy in western countries (Bray et al. 2005; Siegel et al. 2013), as well as in developed cities in China such as Beijing, Shanghai, and Zhongshan. It has supplanted cervical cancer as the leading gynecological malignancy (Siegel et al. 2013, 2014; Gao et al. 2015). With the increasing morbidity and mortality of endometrial carcinoma around the world, there would be social and economic benefit from a screening tool that could be used for early detection, leading to earlier treatment of endometrial carcinoma. Unfortunately, unlike cervical cancer, no effective and low-cost screening program for early detection of endometrial carcinoma has been established at this time (Broso 1995). Curetting is still the standard procedure for evaluating endometrial lesions such as carcinoma and hyperplasia, but this procedure has several deficiencies. Besides of its tendency to cause pain and injury, as well as its cost (Tabata et al. 2001), curetting can only evaluate less than half of the uterine cavity in approximately 60 % of dilatation and curettage (D&C) procedures, which can result in false-negative diagnoses (Kipp et al. 2008). Therefore, there is an urgent need for alternative devices which can be used for early detection of endometrial lesions, especially carcinoma and its precursors. Since the 1970s, several uterine sampling devices which show relatively high sensitivity and specificity for early diagnosis of endometrial lesions have been developed for screening for endometrial lesions (Longacre et al. 1995; Bistoletti and Hjerpe 1993; Vuopala et al. 1989; Tajima et al. 1998; vanHoeven et al. 1996). This review provides an overview of available endometrial devices for sampling and early diagnosis of endometrial lesions, critically evaluates the advantages and deficits of these samplers, and suggests considerations for future development of such devices.

## Methods

A literature search from January 1, 1990 to July 10, 2015 was performed using PubMed for articles about endometrial devices. The keywords used included “aspiration technology for endometrium,” “endometrial aspiration device,” “Pipelle biopsy,” “brush for endometrium,” “brush for endometrial carcinoma,” “Tao Brush”, and “SAP-1 device.” Searches were restricted to human studies and English language publications, and other articles concerning endometrial pathology were excluded. Seventy-four relevant articles on endometrial sampling devices were obtained for our review. Citation lists of retrieved articles were checked to ensure sensitivity of the search methods.

## Results

**Fig. 1:**
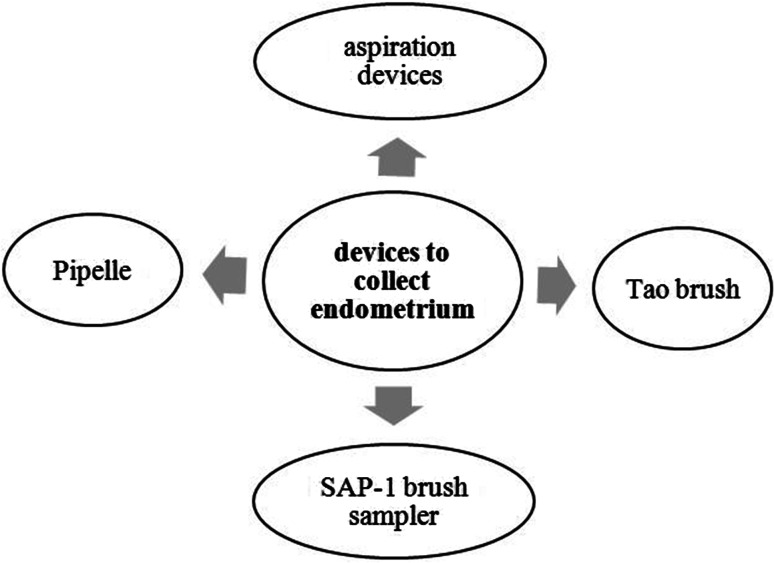
Ways to collect endometrium

The main screening devices for endometrial carcinoma are aspiration devices, Pipelle, Tao Brush, and SAP-1 brush sampler, as outlined in Fig. 1.

### Aspiration devices

Aspiration technology was described by Bela Lorincz, in 1934, as a method with few complications that could be used in an outpatient setting. More recently, aspiration technology has been shown to be a safe, simple, and reliable technique for screening for endometrial lesions (Hemalatha et al. 2006; Kaur et al. 2014; Kawana et al. 2005; Roberts et al. 1994; Rosler et al. 1991; Morse et al. 1982; Niklasson et al. 1981; Poppendiek and Bayer 1981; Masukawa 1981; Smith et al. 1980). Among all of the aspiration devices, the Vabra aspirator is most commonly used for clinical trials to evaluate endometrial lesions. The Vabra aspirator is a metal cannula with a length of 24 cm and an external diameter of 3 mm. At the inner side of the curved ending, it has an aperture of 1.5 × 16 mm. The cannula is connected to a plastic receptacle, which contains a sieve, also of plastic material, to retain the fragments of tissue. After insertion of the cannula into the uterine cavity, the pump is switched on. The two proximal openings in the cannula are covered by an index finger to create negative pressure, while holding the plastic receptacle of the Vabra aspirator. As a result, the uterus is emptied by suction, and then the cannula is withdrawn from the uterus briefly. The procedure is then repeated several times to make sure that the whole interior surface of the uterus is sampled (Lubbers 1977).

In one study, the specimens obtained by aspiration were adequate for cytology in 93 % of cases, among 150 patients (Tripathy and Mahanty 1990). In a symptomatic group of 100 women, a specificity of 88.7 % and a sensitivity of 88.2 % were found, in comparison to histological diagnosis, with only 2 % of the results being false negative. The use of aspiration in endometrial cell sampling also seems to be promising as a screening tool in asymptomatic women (Rosler et al. 1991). In an article by Rodriguez et al., twenty-five patients who were scheduled for hysterectomy were randomly assigned to undergo preoperative endometrial biopsy by Pipelle device (discussed below) (12 patients) or Vabra aspiration (13 patients). The Vabra aspirator was shown to be statistically superior in sampling the percentage of endometrial surface, the mean number of endometrial surfaces, and the mean number of endometrial quadrants (Rodriguez et al. 1993).

Though aspiration sampling could reduce costs compared with curettage, one limitation is that it may be perceived as a procedure for abortion-related treatment in some countries where abortion is illegal (Foster-Rosales et al. 2003). The procedure success rate to collect endometrium in the Vabra was shown to be less than the Pipelle (88.7 % vs. 98.7, *P* = 0.02). Cost-benefit analysis by Naim et al. revealed a higher average cost per patient in the Vabra group compared to the Pipelle group (Naim et al. 2007). Additional limitations of the Vabra aspirator were that in some cases it did not result in adequate endometrium being obtained and did not allow diagnosis of other uterine pathologies, such as endometrial polyps and uterine myoma (Leonardi et al. 1993). As Goldberg et al. reported, the Vabra aspirator could not be inserted in five out of 40 patients because of cervical stenosis (Goldberg et al. 1982). Kaunitz et al. compared the performance of the Pipelle to the Vabra aspirator in 50 patients, and found that the Pipelle obtained more tissue than the Vabra in 28 patients (50 %), and was noted by the clinician to cause less pain in 50 patients (89 %). Forty-seven patients (84 %) stated that biopsy with Pipelle was less painful than with Vabra aspirator (Kaunitz et al. 1988; Wu et al. 2000).

Other aspirators such as Accurette (Kriseman 1982), Isaacs cell sampler (Polson et al. 1984), manual vacuum aspiration (Foster-Rosales et al. 2003; Kitiyodom 2015), and corkscrew (Sierecki et al. 2008) have also been used for evaluating endometrial lesions, but they have not been extensively applied in the clinic because of a lack of supporting data.

### Pipelle

Introduced by Cornier in 1984 (Eddowes et al. 1990), the Pipelle is the most studied biopsy device in the literature (Eddowes et al. 1990; Youssif and Mcmillan 1995; Eddowes et al. 1990; Leng et al. 2013; Fakhar et al. 2008; Elsandabesee and Greenwood 2005; Machado et al. 2003; Dijkhuizen et al. 2000; Sundsbak and Jebsen 1994; Zorlu et al. 1994; Sanam and Majid 2015; Ben-baruch et al. 1994; Leclair et al. 2011). The Pipelle device is 23.5 mm in length, with a polypropylene sheath with an outer diameter of 3.1 mm. Suction is created along a negative pressure gradient when the inner plunger is withdrawn (Leclair et al. 2011). The Pipelle endometrial sampler can be used without cervical dilatation in the outpatient department and causes minimum discomfort.

As compared to traditional D&C, Pipelle sampling is a less time-consuming procedure (Sanam and Majid 2015; Rauf et al. 2014). The specimen satisfaction rate of Pipelle, according to articles from 1994 to 2015, ranged from 73.9 to 100 %. Meanwhile, pathological accuracy was 62.0 to 96.9 % for endometrial lesions (Leng et al. 2013; Fakhar et al. 2008; Machado et al. 2003; Zorlu et al. 1994; Sanam and Majid 2015; Rauf et al. 2014; Gungorduk et al. 2014; Kazandi et al. 2012; Guido et al. 1995; Ben-Baruch et al. 1994), with greater acceptability for patients than D&C (Table 1).

**Table 1:** Review of the literature on Pipelle biopsy for diagnosis of endometrial lesions

Year	References	Cases	Curettage or hysterectomy	Pipelle	Specimen satisfaction (%)	Pathological accuracy (%)
2015	Sanam and Majid (2015)	130	130	130	88.0	94.0
2014	Rauf et al. (2014)	203	101	102	98	–
2014	Gungorduk et al. (2014)	267	189	78	–	62.0
2013	Leng et al. (2013)	200	200	200	93.0	85.0
2012	Kazandi et al. (2012)	82	82	82	93.0	66.0
2008	Fakhar et al. (2008)	100	100	100	98.0	94.0
2003	Machado et al. (2003)	1535	168	1535	73.9	96.9
1995	Guido et al. (1995)	65	65	65	97.0	83.0
1994	Zorlu et al. (1994)	26	26	26	100	95.0
1994	Ben-baruch et al. (1994)	269	97	172	90.6	95.5

Although adequate tissue for histopathologic examination was obtained in slightly fewer cases than for D&C (98 vs. 100 %), the acceptability of the Pipelle was 98 % and of the D&C was 34 % in a study by Raufet al. (Rauf et al. 2014). In a meta-analysis by Dijkhuizen et al. the results of Pipelle sampling were compared with other surgeries such as D&C, hysteroscopy, or hysterectomy between 1996 and 1999, and the conclusion was that the Pipelle was the best device for detecting endometrial lesions in both postmenopausal and premenopausal women, with detection rates of 99. 6 and 91 %, respectively (Fakhar et al. 2008). In the study of Demirkiran et al., 673 patients were evaluated by Pipelle biopsy from October 2007 to November 2009. Compared with pathological examination after hysterectomy, the histological concordance rate was only 67 % for Pipelle biopsy and 70 % for D&C. The sensitivity of Pipelle biopsy and D&C was both 99 %, but Pipelle was easier to perform than D&C for surgeons (Demirkiran et al. 2012). Gungorduk et al. evaluated patients undergoing hysterectomy for various indications via Pipelle endometrial biopsy or D&C from 2009 to 2011. A total of 267 women were included, with 78 women enrolled in the Pipelle group and 189 in the D&C group. The concordance rate with histological diagnosis between Pipelle biopsy and hysterectomy was 62 %, and between D&C and hysterectomy was 67 %. Pipelle biopsy and D&C were equally successful for diagnosing endometrial lesions (Gungorduk et al. 2014). Abdelazim et al. compared the diagnostic sensitivity of Pipelle with D&C in patients undergoing abnormal uterine bleeding; the rate of obtaining adequate endometrium for histological diagnosis was 100 % for D&C and 97.9 % for the Pipelle group. The sensitivity of Pipelle biopsy was 100 % for endometrial hyperplasia, endometrial carcinoma, proliferative, and secretory endometrium, whereas the sensitivity for diagnosing endometritis was 88.9 % (Abdelazim et al. 2013). Pipelle was also shown to be useful in obtaining endometrial tissue for hormonal evaluation (Check et al. 1989), as well as in patients treated with progestin for endometrial hyperplasia (Kim et al. 2015).

However, other reports describe the limitations of the Pipelle biopsy. It may be less efficient than other methods as a screening tool, because only a small proportion of the endometrial surface can be sampled (Batool et al. 1994), and furthermore, the Pipelle biopsy has limited ability to identify focal lesions (Kazandi et al. 2012). In Tanriverdi et al.’s report, 13 patients in the D&C group and 29 patients in the Pipelle group had insufficient tissue, among 127 patients. The authors concluded that Pipelle sampling should be reserved for those patients with only a minimal risk for endometrial carcinoma, hyperplasia, and polyps (Tanriverdi et al. 2004). The sensitivity and specificity of Pipelle in endometrial samplings were compared to fractional curettage in postmenopausal patients and found to be 87.5 and 100 %, respectively. One out of three cases of endometrial adenocarcinoma could not be diagnosed by Pipelle (Bunyavejchevin et al. 2001). Tumors localized to a polyp or a small area of endometrium may go undetected with Pipelle (Guido et al. 1995), and the Pipelle procedure is almost eight times as costly as D&C (Rauf et al. 2014). The Pipelle also showed its limitations for diagnosing endometrial polyps, with a sensitivity of only 60 % in the report by Abdelazim et al. (2013).

### Tao Brush

**Fig. 2:**
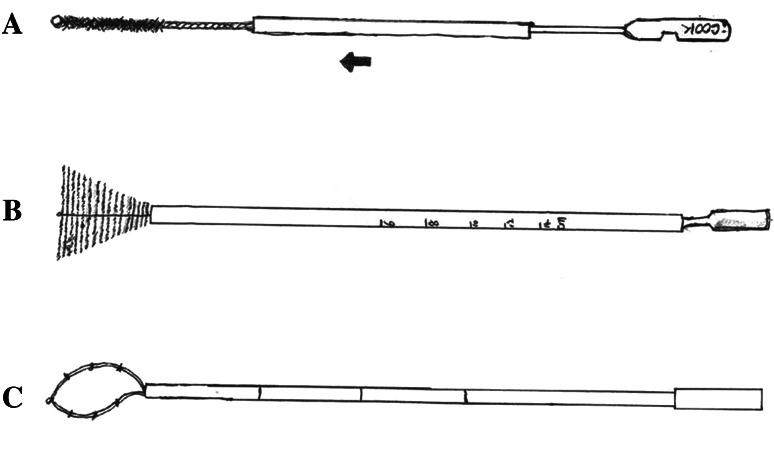
Three types of brushes for endometrial cytology. **a** Tao Brush; **b** Li Brush; **c** SAP-1 endometrial sampler

The Tao Brush was introduced in 1993 and approved by the Food and Drug Administration for general medical use (Tao 1997) (Fig. 2a). To begin collection of endometrial cells, the sheath is pulled back, and then the brush is inserted at the level of the fundus through the cervical canal. The 3.5-cm brush is then rotated 360° 3–5 times to collect endometrial cells. The outer sheath is then pushed back to the tip, and the device is removed from the uterine cavity. The brush is cut off and immersed into cell preservation liquids and sent for cytological assessment and diagnosis (Kipp et al. 2008).

The Tao Brush can be used in an outpatient setting, without the need for anesthetic, as it is simple to use and appears to be well tolerated by women. Specimen satisfaction with the Tao Brush, according to articles from 1997 to 2003, was 89.9 to 100 %, while the pathological accuracy was 91.0 to 96.0 % (Wu et al. 2000, 2003 Maksem et al. 1997; Del Priore et al. 2001) (Table 2).

**Table 2:** Review of the literature on Tao Brush for diagnosis of endometrial lesions

Year	References	Cases	Tao Brush	Curettage or hysterectomy	Specimen satisfaction (%)	Pathological accuracy (%)
1997	Maksem et al. (1997)	100	100	100	100	96.0
2001	Del Priore et al. (2001)	101	101	101	–	95.5
2000	Wu et al. (2000)	200	200	200	95.5	92.5
2003	Wu et al. (2003)	633	633	156	89.9	91.0

There was less specimen insufficiency for diagnosis with the Tao Brush (2 %) than with the Pipelle (12 %). Additionally, the Tao Brush was significantly less painful than Pipelle (*P* < 0.01) (Yang and Wan 2000). In Del Priore et al.’s study, the Tao Brush had 95.5 % sensitivity and the Pipelle had 86 % sensitivity, when correlated with the final diagnosis (Del Priore et al. 2001). The sensitivity and specificity were 100 % for detecting atypical hyperplasia and carcinoma with Tao Brush in 200 cases in the report of Wu et al. (2000). Williams et al. found that adequate samples were significantly more likely to be obtained using the Tao Brush than the Pipelle among 200 high-risk women. A significantly greater proportion of women preferred the Tao Brush to the Pipelle endometrial sampler (Williams et al. 2008).

However, it was difficult using the Tao Brush to distinguish simple hyperplasia without atypia from disordered proliferative endometrium or to diagnose endometrial polyps, according to Wu et al. (2000). Insertion of the Tao Brush was unsuccessful in 20 % of nulliparous women and 8 % of parous women, whereas Pipelle was unsuccessful in 22 % of attempts among nulliparous women compared with 8 % of parous women in Williams’ research. The additional cost of the Tao Brush biopsy compared to the Pipelle biopsy was approximately £100 (Williams et al. 2008). Also, in theory, the Tao Brush has the disadvantage of not collecting enough endometrial cells of the uterine horns because of its round configuration.

### SAP-1 device

The SAP-1 device (Fig. 2b) was patented and received permission to be used in China in 2001. The sheath of this sampler is approximately 3 mm in diameter and 25 cm in length. This protective sheath outside the loop can prevent contamination with cervical and vaginal cells (Wen et al. 2015). To collect endometrial cells, the device is first inserted to the level of the fundus and then the outer sheath pulled back, and then the loop is rotated in a clockwise direction for 15 circles. After collecting enough endometrial cells, the outer sheath is pushed to the tip and the device removed.

The SAP-1 sampler may become a reliable method for screening endometrial carcinoma and its precursors, especially in postmenopausal and asymptomatic women. In the study by Wen et al., adequate specimens for cytology were obtained from 1458/1541 patients (96.3 %) using the SAP-1 sampler. The accuracy of endometrial cytology for diagnosing endometrial carcinoma and its precursors was 92.4 % (sensitivity, 73 %; specificity, 95.8 %; positive predictive value, 75 %; and negative predictive value, 95.3 %) (Wen et al. 2015). However, there have not been enough clinical trials to date supporting the feasibility of the SAP-1 device. Like the Tao Brush, in theory, the SAP-1 device also will not adequately collect cells in the uterine horn.

Other samplers such as the Uterobrush (Fujihara et al. 2006; Iavazzo et al. 2011), Medscand Endorette (Moberger et al. 1998), Cytospat (Antoni et al. 1997), Endopap (Van den Bosch et al. 1996), Tis-U-Trap (Koonings et al. 1990a, b; Frishman and Jacobs 1990), Honest Uterine Brush (Yanoh et al. 2014) have also been used in the clinic, but reports detailing their use appear less often in the literature.

### Li brush

Due to the limitations of the samplers described above, our team invented a new endometrial sampler, named the Li Brush (Fig. 2c), which received a patent in 2014 (ZL.201420720356.8). Compared with other samplers, the Li Brush was designed as an inverted cone, similar in shape to the uterine cavity. In theory, this Brush can collect more endometrial cells than possible with other samplers, especially cells in the uterine horns (Fig. 2), allowing a more accurate diagnosis of endometrial lesions. Clinical trials of the Li Brush have been launched in outpatient and inpatient clinics in the Department of Gynecology of the First Affiliated Hospital, Xi’an Jiaotong University (XJTU1AHCR 2014-007).

## Conclusions and future challenges

All of the devices described herein for evaluating endometrial changes have some disadvantages or limitations for clinical use. It appears that in clinical trials the Tao Brush provides more accurate diagnoses, specimen satisfaction, and pathological accuracy for detecting endometrial lesions. However, sampling insufficiency and costs remain problems that need to be solved for effective screening of endometrial lesions. The devices being developed for endometrial screening should possess the following characteristics. First of all, the screening tool should collect as much endometrial specimen as possible for evaluation and diagnosis, especially cells in the uterine horns. Second, the endometrial specimen should accurately reflect the condition of the uterine cavity to more accurately guide clinical intervention and treatment. Furthermore, the screening tool should be cost-effective in order to be used in a wide range of women for early detection of endometrial lesions, with the goal of improving the prognosis of endometrial carcinoma. Thus, additional efforts should be undertaken to develop an endometrial screening device that could provide more complete histological and cytological information about the uterine cavity, which could be widely used by the female population.

## Abbreviation

D&C: Dilatation and curettage
